# Recessive variants in MYO1C as a potential novel cause of proteinuric kidney disease

**DOI:** 10.21203/rs.3.rs-4183332/v1

**Published:** 2024-04-11

**Authors:** Izzeldin Elmubarak, Shirlee Shril, Bshara Mansour, Aaron Bao, Caroline Kolvenbach, Sherif El Desoky, mohamed shalaby, Jameela Kari, Friedhelm Hildebrandt, Ronen Schneider

**Affiliations:** Boston Children’s Hospital; Boston Childrens Hospital: Boston Children’s Hospital; Boston Childrens Hospital: Boston Children’s Hospital; Boston Childrens Hospital: Boston Children’s Hospital; Boston Childrens Hospital: Boston Children’s Hospital; King Abdulaziz University Faculty of Medicine; King Abdulaziz University Faculty of Medicine; King Abdulaziz University Faculty of Medicine; Boston Childrens Hospital: Boston Children’s Hospital; Boston Childrens Hospital: Boston Children’s Hospital

## Abstract

**Background:**

Steroid-resistant nephrotic syndrome is the second leading cause of chronic kidney disease among patients <25 years of age. Through whole exome sequencing, identification of >65 monogenic causes has rendered insights into disease mechanisms of nephrotic syndrome.

**Methods:**

To elucidate novel monogenic causes of NS, we combined homozygosity mapping with ES in a worldwide cohort of 1649 pediatric patients with NS.

**Results:**

We identified homozygous missense variants in *MYO1C* in two unrelated children with nephrotic syndrome (c.292C>T, p.R98W; c.2273 A>T, p.K758M). We evaluated publicly available kidney single-cell RNA sequencing datasets and found *MYO1C*to be predominantly expressed in podocytes. We then performed structural modeling in molecular viewer PyMol using the super function aligning shared regions within both partial structures of MYO1C (4byf and 4r8g). In both structures, calmodulin, a common regulator of myosin activity, is shown to bind to the IQ motif. At both residue sites (K758; R98), there are ion-ion interactions stabilizing intradomain and ligand interactions: R98 binds to nearby D220 within the Myosin Motor Domain and K758 binds to E14 on a calmodulin molecule. Variants of these charged residues to non-charged amino acids could ablate these ionic interactions, weakening protein structure and function establishing the impact of these variants.

**Conclusion:**

We here identified recessive variants in *MYO1C* as a potential novel cause of nephrotic syndrome in children.

## Introduction

Nephrotic syndrome (NS) is characterized by substantial proteinuria (> 40 mg/m2/hour), leading to hypoalbuminemia, edema, and hyperlipidemia. Approximately 80% of pediatric and young adult patients go into remission with standard steroid therapy, termed steroid-sensitive nephrotic syndrome (SSNS) [1]. Conversely, about 20% of individuals with steroid-resistant nephrotic syndrome (SRNS) are likely to progress to chronic kidney disease (CKD) and ultimately kidney failure [1]. The predominant renal histological feature of SRNS is focal segmental glomerulosclerosis (FSGS), which stands as the second most prevalent cause of kidney failure in the first two decades of life [2].

More than 65 genes have been identified as playing a role in monogenic SRNS, offering valuable insights into the complex mechanisms driving proteinuric kidney disease, SRNS, and podocyte biology [3]. Despite the relative rarity of these genetic variants, their discovery substantially deepens our understanding of podocytopathies. Furthermore, the identification of novel genes associated with SRNS expands the repertoire of genes incorporated into diagnostic gene panels, which are becoming increasingly accessible and utilized in clinical practice.

The glomerular filtration apparatus comprises a three-layered structure consisting of fenestrated endothelium, the glomerular basement membrane, and interconnected podocytes linked through the slit diaphragm. The actin network within podocytes is critical in maintaining foot process integrity, facilitating slit diaphragm turnover, and providing structural support amidst varying glomerular pressures. Genetic variants in genes encoding actin-interacting proteins are prevalent among the monogenic causes of SRNS, underscoring the central role of actin in podocyte physiology [4] [5].

Myosins serve as pivotal actin-based molecular motors, playing important roles in a wide array of cellular functions such as intracellular trafficking, cell adhesion, motility, and maintenance of membrane tension. Among them, MYO1C, a non-muscle myosin motor protein, exhibits high expression levels in glomerular podocytes [6]. It plays a crucial role in the function of the glomerular filter by interacting directly with nephrin and neph1, facilitating their transport to podocyte intercellular junctions [7]. Depletion of myo1c disrupts the localization of these slit diaphragm proteins at the podocyte cell membrane and leads to an edematous phenotype and abnormal podocyte morphology, as evidenced in zebrafish studies [7]. Moreover, podocyte-specific *MYO1C* knockout in mice highlights its critical role in TGF-β-signaling in podocyte disease pathogenesis [6].

Based on our prior investigations, we have established that whole exome sequencing (WES) can uncover a monogenic basis for steroid-resistant nephrotic syndrome (SRNS) in 11–45% of familial cases, often by detecting variants within one of the > 65 published genes [8–10].

Therefore, we hypothesized that previously unidentified single-gene causes of SRNS may be discovered in children and young adults affected by nephrotic syndrome. To investigate this hypothesis, we conducted ES on patients with SRNS/FSGS, leading to the identification of variants in *MYO1C* in two unrelated families.

## Materials and Methods

See Supplementary Materials [10–25].

## Results

To identify potential candidate genes for SRNS, we employed WES on an international cohort of 1649 individuals with NS. We discovered two distinct homozygous missense variants in the MYO1C gene, which encodes the motor protein Myosin 1C, in two unrelated families with proteinuric kidney disease.

**Figure 1:**
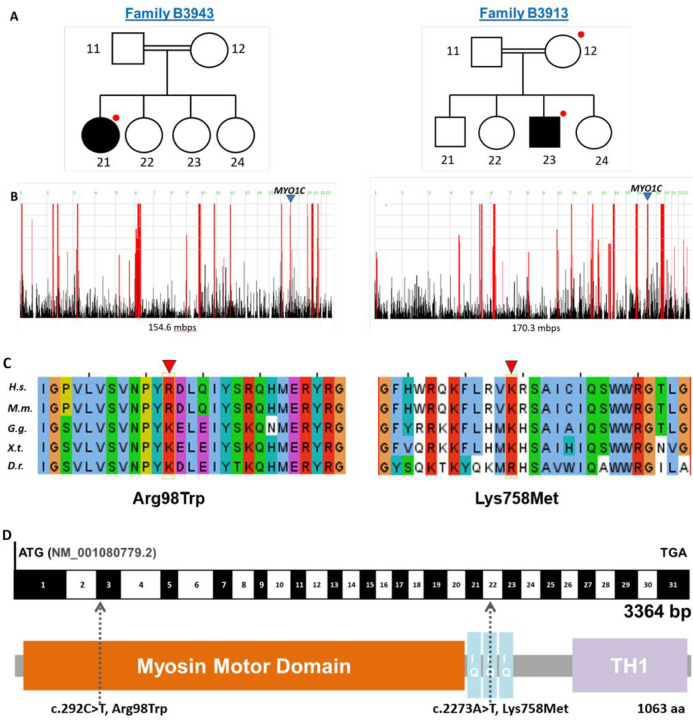
WES identifies biallelic missense variants in the gene MYO1C encoding the motor protein Myosin 1C, in two unrelated families with nephrotic syndrome. (a) Pedigree of index families (B3913 and B3943). Squares represent males, circles females, double line denotes consanguinity, black shading indicates the affected individual, and red dots highlight individuals included in whole exome sequencing. (b) homozygosity mapping depicts high homozygosity in both families and confirms the reported consanguinity. A homozygous peak on chromosome 17 (arrowhead) that includes MYO1C is present in both families. (c) Evolutionary conservation across orthologues of MYO1C. Respective variants are indicated by the arrowhead. (d) Exon structure (black and white) and domain structure (colored) of MYO1C, depicting the location of the two MYO1C variants. Mbps, mega base pairs; aa, amino acids; H.s., Homo sapiens; M.m., Mus musculus; G.g., Gallus gallus; X.t., Xenopus tropicalis; D.r., Danio rerio

In family B3913, a homozygous missense variant c.292C > T (p.R98W) was identified, while in family B3934, a homozygous variant c.2273 A > T (p.K758M) was detected. We confirmed the presence of both variants by Sanger sequencing (Supp Fig.2). Importantly, neither variant was present homozygously in GnomAD, and only 5 and 14 heterozygous variants, respectively, were listed in the database of this control population (n = ~ 200,000 individuals) [17]. Evolutionary conservation analysis indicates that at the modified amino acid position, a positively charged amino acid—either arginine or lysine—is conserved across species down to Danio rerio. Two out of three bioinformatic prediction programs (SIFT, Mutation Taster and Polyphen-2) classified both variants as pathogenic (Table 1). The identified variants were located within well-defined functional domains, specifically the N-terminal myosin motor domain and the calmodulin-binding IQ domain, respectively (Fig. 1D). The affected individual in family B3913 is an Arabic male who developed steroid-dependent nephrotic syndrome at the age of 3. He has no extrarenal manifestation and has not had a kidney biopsy. In family B3943, an Arabic male developed steroid-resistant nephrotic syndrome at the age of 13, with no extrarenal manifestation, and a kidney biopsy revealed membranoproliferative glomerulonephritis. Consanguinity was reported in both families, and it was confirmed with homozygosity mapping showing runs of homozygosity spanning 154.6 Mb and 170.3 Mb respectively. Notably, the candidate variants in MYO1C coincided with a peak indicating a region of homozygosity by descent (Fig.1).

**Figure 2:**
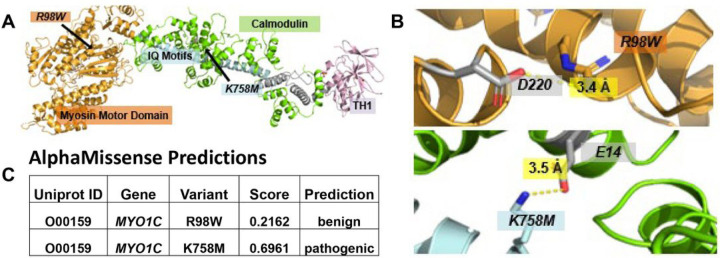
Crystallized structures of MYO1C (PDB: 4byf, 4r8g). Structural modeling was performed in molecular viewer PyMol using the super function aligning shared regions within both partial structures. In both 4byf and 4r8g structures, calmodulin, a common regulator of myosin activity, is shown to bind to the IQ motif. (A) Complete MYO1C protein structure modeled next to calmodulin. (B) Close-up view of molecular residue interactions at both mutation sites. At both residue sites (K758, R98), there are ion-ion interactions stabilizing intradomain and ligand interactions: R98 binds to nearby D220 within the Myosin Motor Domain and K758 binds to E14 on a calmodulin molecule. Mutations of these charged residues to non-charged amino acids could ablate these ionic interactions, weakening protein structure and function. (C) AlphaMissense analyzes the effects of missense variants on protein tertiary structure. It predicts K758M to be a pathogenic mutation with a score of 0.69

We examined kidney single-cell RNA sequencing datasets available publicly and identified strong podocyte expression of MYO1C in comparison to positive (nephrin and podocin) and negative controls (ACTA2) ([26]) (Supp Fig.1). Next, using the molecular viewer PyMol software, we conducted structural modeling to align shared regions within both partial structures of MYO1c. (4byf and 4r8g), aligned with the common regulator of myosin activity, calmodulin (Fig.2). Calmodulin was observed to bind to the IQ motif in both structures. At residue sites (K758, R98), ion-ion interactions were discerned, stabilizing intradomain and ligand interactions: specifically, R98 forms a bond with nearby D220 within the Myosin Motor Domain, while K758 interacts with E14 on a calmodulin molecule. We analyzed the effects of the variants on protein tertiary structure utilizing the AlphaMissense software [27]. The R98W exchange in the myosin domain (Fig.2A) was predicted to be benign, whereas K758M, which interacts with glutamate E14 on calmodulin was predicted to ablate this ionic bond and was deemed likely pathogenic (Fig.2C).

## Discussion

In this study, we identified two homozygous missense variants in the gene *MYO1C* in two unrelated families with proteinuric kidney disease. Reported consanguinity and measured homozygosity in both families suggest a recessive cause of disease. Indeed, homozygous *MYO1C* variants were detected in peaks on homozygosity mapping. Both variants are missense alterations resulting in a positively charged amino acid, arginine or lysine, substituted by a neutral amino acid. Evolutionary conservation, pathogenicity prediction by in-silico programs and 3D structure prediction programs, and position within protein domain structures, support the pathogenic role of these *MYO1C* variants.

The majority of causative SRNS genes exhibit high level expression in glomerular podocytes, strongly suggesting podocytes to be the main site of injury in NS and underscoring its crucial role in upholding the filtration barrier. *MYO1C* is predominantly expressed in podocytes as evidenced by single-cell RNA sequencing datasets [26] (Supp Fig.1). This supports a potential pathogenic role of MYO1C in SRNS. Moreover, 3D structural modeling revealed ion-ion interactions at both residue sites (K758, R98), which likely play an important role in stabilizing intradomain and ligand interactions. Modifying these charged residues to non-charged amino acids could potentially disrupt these ionic interactions, leading to a weakening of protein structure and its function. Published functional data further supports the pivotal role of MYO1C in podocyte physiology. MYO1C was shown to be a direct interactor of the podocyte slit diaphragm structural proteins, nephrin and neph1, and a mediator of their transport to the podocyte intercellular junction [7]. Arif et al. demonstrated abnormal developmental phenotype of *myo1c* knockout zebrafish, characterized by pericardial edema and dilated renal tubules [6]. Subsequent analysis of the glomerular ultrastructure in *myo1c* depleted zebrafish revealed absence of the slit diaphragm and abnormal podocyte morphology. Furthermore, their research highlighted the central role of *Myo1c*-mediated regulation of TGF-b in the pathogenesis of podocyte injury, as evidenced by findings from a *Myo1c* knockout mouse model [7].

**Figure 3:**
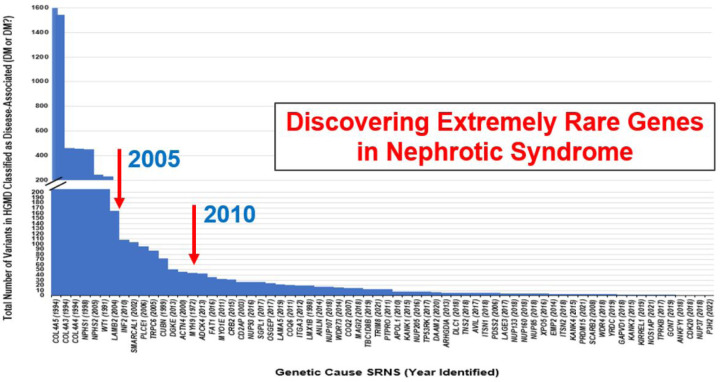
The number of disease-causing alleles currently listed in the HGMD database for the 67 monogenic causes of steroid-resistant nephrotic syndrome. The more frequently mutated genes were discovered first, such as nephrin, podocin, WT1 and LAMB2. Genes like INF2 and PLCE1 were discovered before 2010, and thereafter newly discovered genes were extremely rare

Although the number of known genes associated with SRNS has increased from 27 to 69 over the last decade, the impact on the solve rate has been limited [Supp Table 1]. This can be explained by the rarity of the newly discovered genes as shown in Fig.3. Analyzing this chart reveals a discernible trend: there appears to be a correlation between the year a gene was discovered as being causative of SRNS, and its prevalence. Notably, the genes implicated latest as monogenic causes of SRNS are exceptionally rare, being present in only a few families.

Based on our prior experience, an initial discovery of a rare variant becomes the basis for identifying additional variants in the candidate gene. With sufficient genetic evidence, usually when four different families with distinct alleles in the same gene are discovered, functional studies are, then, warranted to support the biological impact of disease-causing variants. Nevertheless, when lacking this threshold, we still find it crucial to report these discoveries, once genetic evidence, computational methods, and existing literature strongly support a potential role of the gene-product in the pathogenesis of SRNS. For instance, KANK1 and CRB2, initially identified by our lab in one and four families respectively [28, 29], have now been reported in seven and thirty-one families in Human Gene Mutation Database, indicating the evolving landscape of genetic discoveries in nephrotic syndrome.

In conclusion, we here present variants in *MYO1C* in two families, which may point to *MYO1C* as a potential new candidate gene for SRNS. Discovery of further families with NS who carry variants in the *MYO1C* gene, together with functional evidence to support their pathogenicity, are necessary to assess the role of *MYO1C* as a candidate gene for SRNS.
